# Patients’ Insight of Interpreting Prescriptions and Drug Labels - A Cross Sectional Study

**DOI:** 10.1371/journal.pone.0065019

**Published:** 2013-06-03

**Authors:** Muhammad Junaid Patel, Muhammad Shoaib Khan, Farheen Ali, Zehra Kazmi, Talha Riaz, Safia Awan, Ayesha L. Sorathia

**Affiliations:** Department of Internal Medicine, Aga Khan University, Karachi, Pakistan; Universidad Peruana Cayetano Heredia, Peru

## Abstract

**Background:**

Errors in consuming drugs are associated with significant morbidity and mortality, besides an impact on the already overburdened health-care system. Misunderstanding drug labels and prescriptions plays an important role in contributing to adverse drug events.

**Objective:**

To evaluate abilities to understand prescriptions and drug labels among patients attending tertiary care hospital in Karachi.

**Methods:**

A cross sectional study was conducted at the Aga Khan University Hospital (AKUH), from January to March 2009. After informed consent, 181 adult patients and their healthy attendants were interviewed at AKUH using a standardized questionnaire, which ascertained patient demographics, factors that might increase exposure to health-care personnel as well as the basic knowledge and understanding of prescriptions and drug labels.

**Results:**

Out of 181, majority 137(76%) had received graduate or post-graduate degrees. 16 (9%) had received no formal education; of which all were females and 89(84%) of the total females were housewives. Overall, 130(72%) followed only a single doctor’s prescription. Majority failed to understand various medical terminologies related to dosage. In the high literacy group, 45(33%) understood once daily OD (p = 0.003), 27(20%) thrice daily TID (p = 0.05), 29(21%) twice daily BD (p = 0.01), 31(23%) thrice daily TDS (p = 0.002) and 43(31%) as needed SOS (p = 0.003) as compared to the group with no formal education, who were unable to comprehend the terms. The most common reason for using more than one prescription was decreased satisfaction with the doctor in 19(39%) and multiple co-morbids as responded by 17(35%) of patients. Knowledge regarding various medical terminologies used for dosage and routes of drug administration were also understood more frequently among the English medium respondents. The elderly identified medicine through color (47%, p<0.001), and were less likely to understand drug indications (p = 0.05) compared to younger subjects.

**Conclusion:**

Understanding of drug prescriptions is alarmingly low in the community, even amongst the educated. Care givers need to revisit this often ignored aspect of patient care.

## Introduction

Poor understanding of prescriptions and difficulty in reading labels is not uncommon and may lead to an adverse drug event (ADE). [1], [2], [3] ADEs can be caused by the drugs themselves, or result from associated factors such as erroneous prescriptions, supply and drug monitoring. [1] Worldwide, ADEs or drug related injuries account for a large number of hospital admissions annually. [4], [5] In a recently published study from Karachi, 38% of patients admitted to a tertiary care center with drug toxicities were secondary to accidental overdose. [2] These adverse events can occur in both in-patients and out-patients. [1], [6] Errors in taking medications can have high morbidity and mortality rates which significantly impacts the already burdened health-care system. Very importantly, it is possible to prevent many of these events. [2]

A number of factors can contribute to drug related adverse events; the lack of basic health literacy being one of them. [7] Health literacy is defined by the American Medical Association (AMA) as “A constellation of skills, including the ability to perform basic reading and numerical tasks required to function in the health care environment”. [8] Studies have also shown that patients who are uneducated and those with multiple medication prescriptions are more prone to make errors [7]. In addition, vague or complex instructions and distracting drug labels also contribute to the misinterpretation of medical prescriptions and to possible drug related adverse events. [9]

The objective of this study was to assess the abilities to understand prescriptions and drug labels in the local population and the factors responsible for it. To the best of authors’ knowledge, no such study has been conducted in Pakistan. We selected a setting that is one of the more well-equipped tertiary care centres of the country. At this centre clinics are well organized with appropriate time slots given to patients. In addition, every clinic is managed by trained nursing staffs that liaise with the patients as needed. There is also a well established pharmacy system in place, where pharmacists double checks physician’s orders. Medications are then dispensed to the patients with both verbal and written instructions. The premise therefore is that patients receiving care in this institution should have adequate knowledge regarding the medications being prescribed to them.

## Methodology

### Study Setting and Population

A cross sectional study was conducted at the Aga Khan University Hospital (AKUH) from January to March 2009. Convenience sampling method was used to select patients for data collection who visited various specialty and subspecialty clinics of the outpatient department. The interview was conducted after informed consent.

We included adult patients (>18 years old) who were able to comprehend English and/or Urdu and were willing to respond to the questionnaire. Patients unable to give informed consent were excluded from the study.

### The Questionnaire

The questionnaire was developed by a group of physicians in the Department of Medicine and the Research fellows and Epidemiologists affiliated with the department of Medicine. It was then reviewed with the research associates who were responsible for the data collection. The questionnaire addressed patient demographics, factors that might increase exposure to health-care personnel as well as assessed the basic knowledge and understanding of prescriptions and drug labels. Every possible effort was made to reduce any bias by discussion among other researchers. The questions were structured in a manner so as to avoid any ambiguity. The research officers were either graduates (doctors) or undergraduate medical students of advance levels. Multiple preparatory sessions were conducted to discuss various aspects of the questionnaire to reduce interviewer’s bias, followed by a pilot test before conducting this study. The questionnaire was not modified after the pilot study and the results of the pilot study were included in the final results. Ethical approval was obtained from the Aga Khan University (AKU) Ethics Committee. The questionnaire addressed patient demographics, factors that might increase exposure to health-care personnel as well as assessed the basic knowledge and understanding of prescriptions and drug labels.

### Sample Size Estimation and Data Collection

There is no published literature available from Pakistan to determine the knowledge and understanding of prescriptions and drug labels amongst the patient population, therefore with 95% confidence level and 10% bound on error of estimation, a sample of 97 adults was required when p was taken as 0.5. Adding an additional 10% for non-response subjects, a final sample size of 107 study participants was determined. To identify the factors associated with understanding of prescriptions, the sample size for the most important factor (literacy) was estimated. A sample size of at least 96 was estimated, using the prevalence of literacy as 45% and a significance level of 0.05, and a power of 80%. Therefore, a minimum sample of 107 adults was targeted to cover both the objectives.

### Statistical Analysis

All analyses were conducted using the Statistical Package for Social Science **SPSS** (Release 16.0, standard version, copyright © SPSS; 1989-02) All p-values were two sided and considered statistically significant if <0.05. Descriptive statistics were calculated for each variable. Results were expressed as mean ± standard deviation for continuous variables and number (percentage) for categorical data. Chi-square test was used to evaluate the association between literacy, demographic data and other variables.

For the sub group analysis, patients were divided into three categories based on their level of education: **Group 1** were those who had no formal education, **Group 2** had matriculation or high school equivalency as maximum level of education and **Group 3** comprised of patients with graduate and post graduate degrees. This group was further divided and analyzed according to their medium of education i.e; English vs. Urdu.

To assess univariate associations between the outcome and potential predictors, odds ratios (ORs) and their 95% confidence intervals (CIs) were computed by logistic regression analysis. All significant factors on univariate analysis were considered for inclusion in the multivariable logistic model (Table 1).

**Table 1:** Univariate and multivariate analysis of factors associated with understanding prescriptions and drug labels among higher educated participants.

Characteristics	Less than 12 yearsof education = 58	More than 12 yearsof education = 107	p value	aOR ratio[95% CI]	p value
Gender					
Male	15(25.9)	60(56.1)	<0.001	2.45[1.13–5.32]	0.02
Female	43(74.1)	47(43.9)		Reference	
Medium of education					
English	15(25.9)	72(67.3)	<0.001	4.12[1.93–8.81]	<0.001
Urdu	43(74.1)	35(32.7)		Reference	
Do you buy your drugthrough prescription					
Always	38(66.7)	40(37.7)	0.003	–	NS
Most of the time	14(24.6)	38(35.8)			
Sometimes	4(7)	26(24.5)			
Never	1(1.8)	2(1.9)			
Do you understandyour prescription					
Always	18(31)	59(55.1)	0.001	–	NS
Most of the time	11(19)	25(23.4)			
Sometimes	17(29.3)	17(15.9)			
Never	12(20.7)	6(5.6)			
Do you understandmedical terminology					
TDS					NS
Yes	3(5.2)	28(26.2)	0.001	–	
No	55(94.8)	79(73.8)			
IM					
Yes	13(22.4)	49(45.8)	0.004	2.49[1.13–5.49]	0.02
No	45(77.6)	58(54.2)		Reference	

## Results

181 subjects were included in the study. Mean age of subjects was 46 years (SD, 14.07; range, 20 to 80 years). There were 16(9%) patients in Group 1, 28(16%) in Group 2 and 137 (76%) in Group 3(Table 2). For understanding their prescriptions, 138 (76%) respondents consulted their doctor, 25 (14%) relied on self-assessment and 16 (9%) approached the pharmacists and nurses. Subjects in Group 1 were more likely to seek help when taking guidance about their drug prescriptions (94%) as compared to Group 2 (82%). Group 1 also showed a higher trend of buying drugs through prescriptions i.e., 12(75%). Overall, 130(72%) followed only a single doctor’s prescription. The most common reasons for using more than one prescription was lack of satisfaction with their doctor which was in 19(39%) patients, and having multi-system problems, in 17(35%). Overall the common co-morbid conditions included hypertension (27.1%), arthritis 20 (11%), diabetes mellitus 30 (16.6%), Ischemic Heart Disease 11 (6.1%), asthma 7 (3.9%), and chronic renal failure 6 (3.3%).

**Table 2:** Demographics of Patient Population.

Characteristics	Total n = 181	Literacy Level			
		Group 1	Group 2	Group 3	P value
**Gender**					
Male	75 (41)	0	6(21)	69(38)	<0.001
Female	106(59)	16(100)	22(79)	68(37)	
**Medium of Education**					
English	87(53)	0	6(21)	81(45)	<0.001
Urdu	73(44)	0	20(71)	54(30)	
Others	5(3)	0	22(7)	3(2)	
**Co-morbidities**					
Yes	104(58)	15(94)	20(74)	69(38)	0.001
No	77(42)	1(6)	8(29)	68(37)	
**Occupation**					
Professional	58(32)	0	2(7)	56(31)	<0.001
Govt. service	17(9)	0	1(4)	16(9)	
House wife	89(49)	16(100)	21(75)	52(29)	
Retired/unemployed	17(9)	0	4(14)	13(7)	

**Figure 1:**
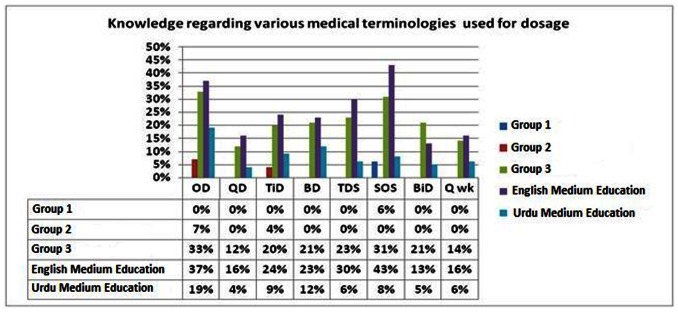
Knowledge regarding various medical terminologies used for dosage.

**Figure 2:**
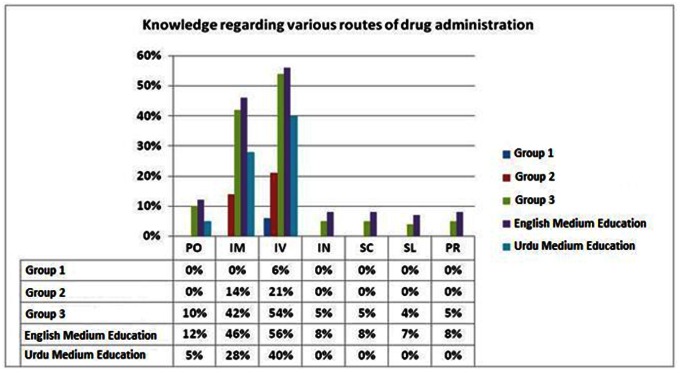
Knowledge regarding various routes of drug administration.

The majority of patients failed to understand various medical terminologies related to the dosage (Figure 1). In addition, poor understanding was observed for the commonly used medical terminologies related to various routes of administering a drug amongst all three groups (Figure 2).

**Figure 3:**
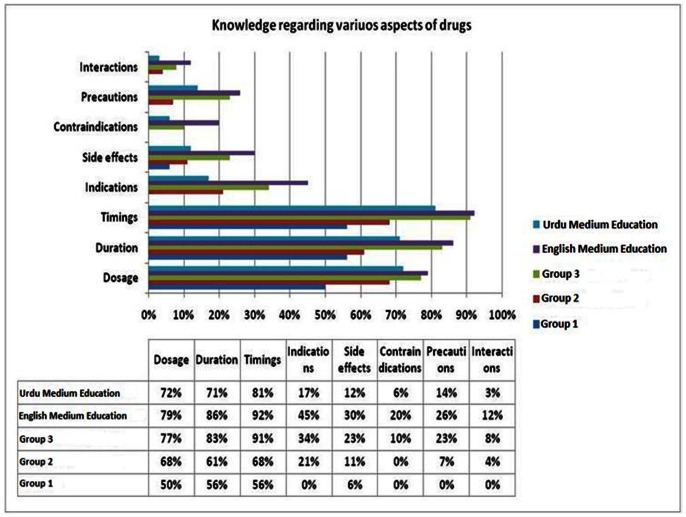
Knowledge regarding various aspects of drugs.

A total of 139(77%) patients always knew the duration of their prescribed drug. Of these, 113(83%) were from Group 3 (p = 0.05). Of 152(84%) respondents who always knew the timing of taking each medication, 124(91%) were from Group 3 (p<0.001). Only 46(34%) of this group always understood the indications (p = 0.003), 32(23%) the side effects (p = 0.02), 32(23%) the precautions (p = 0.003), and 22(10%) the contra-indications (p = 0.004) (Figure-3).

Knowledge regarding various medical terminologies used for dosage and routes of drug administration were understood in a greater proportion of respondents with English as a medium of education compared to with Urdu as a medium of education. Similar trend was observed for the terms commonly used by physicians while writing prescriptions, indications, interactions, side effects, contraindications and the precautions of their prescribed drugs (Figure 1).

One interesting finding was the almost uniform inability to understand physicians’ hand writing. Overall, 151(83%) respondents identified their drugs through labels; 122(89%) in Group 3, 23(82%) in Group 2 and 6(38%) in Group 1 used labels as a tool for identification of their drugs (p<0.001). 49(56%) respondents with English as a medium of education always read and tried to understand their prescriptions (p = 0.01) while only 12(14%) always understood doctors’ writing on the prescription (p = 0.03).

## Discussion

Pakistan is a developing country with limited health care resources and a literacy rate of only 45%. Most medications are bought over the counter without a doctor’s prescription from pharmacies called “medical stores” which are mostly run by non-pharmacists. There is a paucity of places where a proper pharmacy system exists and these are mainly hospital based. [10].

The sample of our study was selected from out -patient clinics as the authors believe that misinterpretation of prescription drugs and drug labels are far more common in ambulatory patients than in hospitalized patients. [2], [9], [11] The reasons for this hypothesis were that ambulatory patients are more likely to self-medicate, the contact with physicians is intermittent and communications regarding any problems these patients may face as far as drug prescriptions are concerned is infrequent. [2] More than fifty percent of our respondents were women, where all of the housewives had no formal education.

Patients’ lack of knowledge regarding prescriptions and drug labels is an important concern. [12] We specifically targeted the basic knowledge regarding prescription drugs and drug labels like dosage, duration, timing, indications, interactions, side effects, contraindications and precautions. We concluded that majority of our selected population has very little information regarding drugs prescribed to them.

It was previously observed in studies that people with high socioeconomic status or higher education level have a better understanding of prescription drugs and drug labels thus leading to lesser incidence of adverse drug events. [9], [12] However; in our study we observed that inability to understand and interpret prescriptions was prevalent across all educational levels. We also observed that patients with multiple sources for understanding prescriptions were more aware than those who used a single source. Patients who received their formal education in English were more aware of different drug aspects and medical terminologies as compared to those who had Urdu language as their medium of education. Age has been identified as a factor in the misunderstanding and misinterpretation of prescription drugs and drug labels. [11] Our results also support this finding in that various aspects of drug usage like dosage, duration and timings etc. were better understood by the younger respondents.

Misunderstanding of drug prescriptions and labels occur when the instructions are unclear and needlessly difficult. [9] This also caused errors at the pharmacist level. [5] One limitation in our study was that majority of the participants were non-English speaking (dominant Urdu speaking population), therefore automatically rendering them unable to read the prescription, and those who understood spoken English, yet were unable to interpret the language of a written prescription. This issue was compounded by abbreviations used in the prescriptions. Also, physician hand writing was not legible enough to be understood even by those with higher education and thus was seen as an obstacle for understanding prescriptions in all groups.

An important finding of this study was that majority of the study population consulted their doctors for understanding the prescriptions. Half of them bought their medications through prescriptions which is higher than previously thought. Interestingly this trend was seen more in those with lower levels of education supporting results from other studies. [13] This emphasizes the fact that an intervention at the level of the physicians may in fact improve patient’s understanding of prescriptions.

## Limitations

Our study group is more privileged in terms of overall education, socio-economic status and availability of health care facilities as compared to the general population of Pakistan and hence selection bias cannot be overlooked. Even though Urdu is the national language, they also speak a variety of other dialects including Punjabi, Pashto, Sindhi, and Balochi etc. As mentioned there is no formal pharmacy system that exists in the country, whereas AKUH has a formal pharmacy system and patients cannot buy medications without prescriptions. This study was therefore done on a population that may not be truly representative of the larger community so external generalizability is questionable. We believe this study needs to be done in different cities of Pakistan on a bigger scale to elicit more relevant results. We also believe that the patients visiting AKUH are skewed towards higher socio-economic status (compared to the general population), hence the other cities are likely to report even lesser comprehension rates than those reported in this study.

### Conclusion

Our study identifies that the majority of the respondents had an alarmingly low level of understanding of prescriptions. Patients with lower levels of literacy seek more help for understanding their prescription. Various drug modalities were better understood in the higher literacy group as compared to the lower literacy groups. Poor understanding of the prescription due to doctor’s hand writing was prevalent regardless of the level of education. The role of physicians in this regard is undeniable.

## Recommendations

Our research suggests that majority of the study population was unable to understand and interpret basic health information and services required to make appropriate health decisions. We suggest that the complex medical terminologies should be avoided from the prescriptions. A better doctor-patient relationship is essential in our set-up. Integrated efforts should be made by the doctors, nurses, pharmacists, patients and the family members. We need more awareness in this crucial aspect of medical management as well as more research on this particular issue.

Since the findings of the study cannot be generalized to the entire population, therefore, a multicenter study targeting all the strata of hospitals should be conducting before safely generalizing the results to entire population.

## References

[1] Gurwitz JH, Field TS, Harrold LR, Rithschild J, Debellis K, et al (2003) Incidence and preventability of adverse drug events among older persons in the ambulatory setting. JAMA 289: 1107–16. 12622580 10.1001/jama.289.9.1107

[2] Gandhi TK, Weingart SN, Borus J, Seger AC, Peterson J, et al (2003) Adverse Drug Events in Ambulatory Care. N Eng J Med 348: 1556–64. 10.1056/NEJMsa020703 12700376

[3] Lazarou J, Pomeranz BH, Corey PN (1998) Incidence of adverse drug reactions in hospitalized patients: A Meta analysis of prospective studies. JAMA 279(15): 1200–1205. 9555760 10.1001/jama.279.15.1200

[4] Patel MJ, Shahid M, Riaz M, Kashif W, Ayaz SI, et al (2008) Drug overdose: a wake up call! Experience at a tertiary care centre in Karachi, Pakistan. J Pak Med Assoc; 58 (6): 298–301. 18988386

[5] Gupta AK, Cooper EA, Feldman SR, Fleischer AB Jr, Balkrishnan R (2003) Analysis of factors associated with increased prescription illegibility: Results from National Ambulatory Medical Care Survey, 1990–1998. Am J. Manag. Care 9(8): 548–52. 12921232

[6] Hanlon JT, Schmader KE, Koronkowski MJ, Weinberger M, Landsman PB, et al (1997) Adverse drug events in high risk older outpatients. J Am Geriatr Soc 45: 945–8. 9256846 10.1111/j.1532-5415.1997.tb02964.x

[7] Safeer RS, Keenan J (2005) Health Literacy: The gap between physicians and patients. Am Fam Phy 72: 463–468. 16100861

[8] Authors not listed (1999) Health literacy: Report of the Council of Scientific Affairs. Committee on Health Literacy for the Council on Scientific Affairs, American Medical Association JAMA 281: 552–7. 10022112

[9] Wolf MS, Davis TC, Shrank W, Rapp DN, Bass PF, et al (2007) To err is human: Patient misinterpretations of prescription drug label instructions. Pat Educ Counsel 67: 293–300. 10.1016/j.pec.2007.03.024 17587533

[10] Labour Force Survey 2007–2008, Government of Pakistan Statistics Division, Federal Bureau of Statistics, Pakistan. Available: www.statpak.gov.pk/fbs/content/labour-force-survey-2007-08. Accessed 2012 Oct 20.

[11] Philips DP, Christenfeld N, Glynn LM (1998) Increase in US medication error deaths between 1983 and 1993: Research letter; The Lancet 28. 351(9103): 643–4. 10.1016/S0140-6736(98)24009-8 9500322

[12] Davis TC, Federman AD, Bass PF 3rd, Jackson RH, Middlebrooks M, et al (2008) Improving Patient Understanding of Prescription Drug Label Instruction. J Gen Intern Med. 20 January 24(1): 57–62. 10.1007/s11606-008-0833-4 PMC2607498 18979142

[13] Davis TC, Wolf MS, Bass PF 3rd, Middlebrooks M, Kennen E, et al (2006) Low Literacy Impairs Comprehension of Prescription Drug Warning Labels J Gen Intern Med. 21(8): 847–851. 10.1111/j.1525-1497.2006.00529.x PMC1831578 16881945

